# Effectiveness of Onabotulinumtoxin-A in Patients with Chronic Migraine and Previous CGRP-mAb Treatment Failure: A Multicentre 6-Month Real-World Experience

**DOI:** 10.3390/toxins18080342

**Published:** 2026-08-04

**Authors:** Marcello Silvestro, Maria Albanese, Ilaria Orologio, Luigi Francesco Iannone, Francesca Pistoia, Stefania Battistini, Gianluca Avino, Martina Petracca, Marina Romozzi, Raffaele Ornello, Diego Centonze, Simona Sacco, Antonio Russo, on behalf of the Italian Headache Registry (RICe) Study Group

**Affiliations:** 1 Inter-Departmental Program of Headache Medicine and Primary Pain Syndromes, Department of Advanced Medical and Surgical Sciences, University of Campania “Luigi Vanvitelli”, 80138 Naples, Italy; marcello.silvestro@unicampania.it (M.S.); ilaria.orologio@unicampania.it (I.O.); 2 Department of Systems Medicine, University of Rome Tor Vergata, 00133 Rome, Italy; maria.albanese@ptvonline.it (M.A.); centonze@uniroma2.it (D.C.); 3 Unit of Neurology, IRCCS Neuromed, 86077 Pozzilli, Italy; 4 Department of Biomedical, Metabolic and Neural Sciences, University of Modena and Reggio Emilia, 41121 Modena, Italy; luigifrancesco.iannone@unimore.it; 5 Department of Biotechnological and Applied Clinical Sciences, University of L’Aquila, 67100 L’Aquila, Italy; francesca.pistoia@univaq.it (F.P.); raffaele.ornello@univaq.it (R.O.); simona.sacco@univaq.it (S.S.); 6 Department of Medical, Surgical and Neurological Sciences, University of Siena, 53100 Siena, Italy; battistinis@unisi.it; 7 Neurology Unit, Ospedale Santo Stefano, USL Toscana Centro, 59100 Prato, Italy; gianluca_avino@yahoo.it; 8 Fondazione Policlinico Universitario Agostino Gemelli IRCCS, Università Cattolica del Sacro Cuore, 00168 Rome, Italy; martina.petracca@policlinicogemelli.it (M.P.); marina.romozzi@guest.policlinicogemelli.it (M.R.)

**Keywords:** onabotulinumtoxin-A, chronic migraine, resistant migraine, CGRP monoclonal antibodies

## Abstract

Background: A substantial proportion of patients with chronic migraine (CM) with previous failures among oral standard-of-care migraine medications do not respond to monoclonal antibodies targeting the calcitonine gene related peptide pathway (CGRP-mAbs), leaving limited evidence-based options for subsequent preventive strategies. The present multicentre real-world study evaluated the effectiveness of onabotulinumtoxin-A (BoNT-A) in CM patients with previous CGRP-mAb failure and multiple prior oral preventive failures. Methods: This prospective, observational, non-randomized multicentre study enrolled patients with CM who had failed ≥3 classes of oral preventive treatments and ≥1 CGRP-mAb due to lack of efficacy or intolerance. Participants received BoNT-A according to the PREEMPT “follow-the-pain” paradigm every three months and were followed for 6 months. Co-primary outcomes were change in monthly headache days (MHD) after three and six months and ≥50% MHD responder rates at both time points. Results: Fifty-three patients were analysed (85% female, aged 48.5 ± 15.2 years, range 20–73). Compared to the baseline, at the third month and sixth month, MHD decreased from 22.83 (SD 6.74) to 15.38 (SD 7.91; *p* < 0.001) and 14.55 (SD 9.35; *p* < 0.001), respectively. The percentages of participants achieving the response status in MMD at the third month and sixth month were 30% and 45%, respectively. In the third and sixth months, 22 patients (41.5%) and 27 patients (50.9%), respectively, converted from chronic to episodic migraine, while the prevalence of medication-overuse headache decreased from 81.1% of patients at baseline to 45.3% and 43.4%, respectively. Migraine-related impact and disability scores improved over follow-up, alongside improvements in anxiety and depressive symptoms and in migraine-specific quality of life. Conclusions: The present findings support the use of BoNT-A in difficult-to-treat patients with CM following CGRP-mAb failure and provide further evidence that its therapeutic effects may rely on mechanisms that are at least partially distinct from, and potentially complementary to, those of CGRP-targeted therapies.

## 1. Introduction

Chronic migraine (CM) affects approximately 1–2% of the general population and represents one of the most disabling neurological disorders worldwide [1,2,3]. It is a major contributor to disability-adjusted life years (DALYs), particularly among young and middle-aged adults [4,5]. Beyond its clinical burden, CM is associated with substantial socioeconomic costs resulting from increased healthcare resource utilization, reduced work productivity, and impaired quality of life, highlighting the importance of effective preventive interventions and optimized management strategies [5,6]. Over the past decade, migraine prevention has undergone a profound therapeutic evolution with the introduction of mechanism-based preventive treatments [7,8]. Among these, monoclonal antibodies targeting the calcitonin gene-related peptide (CGRP-mAbs) pathway—by binding either the ligand or its receptor—have consistently demonstrated high preventive efficacy in randomized controlled trials, with subsequent real-world studies confirming their effectiveness in both episodic migraine and CM, including patients with previous failures of conventional preventive therapies [9]. It is important to emphasize that despite CGRP-mAbs’ effectiveness, a substantial proportion of patients, estimated between 20% and 40%, fail to respond in clinical practice. The management of these patients remains particularly challenging, given their history of multiple failures of oral preventive therapies, as required by current regulations for access to CGRP-mAbs therapies in Italy [10,11]. While several studies have established the efficacy of CGRP-mAbs in patients with prior failure of onabotulinumtoxin-A (BoNT-A) [12,13,14], to date, only a few real-world studies on a small cohort of patients with migraine are available supporting the reverse sequence [15]. Although at first glance this strategy may appear counterintuitive, as both treatments ultimately modulate the CGRP pathway through different sites of action, they differ substantially in their underlying mechanisms. BoNT-A acts presynaptically on C-fibres by inhibiting the vesicular release of several pronociceptive neuropeptides, including CGRP, PACAP, and VIP [16,17]. The broader inhibitory effect on neuropeptide release, together with distinct sites of action, suggests that BoNT-A may exert preventive efficacy even in patients who have not responded to specific blockade of the CGRP pathway (regardless of receptor or ligand itself). Thus, rather than being redundant, the two different mechanisms of action may provide a rationale for exploring BoNT-A as an option in CGRP-mAbs non-responders. Although the combined use of BoNT-A and CGRP-mAbs has been shown to provide additive or potentially synergistic effects [18,19], it may also be effective in evaluating the therapeutic benefit of BoNT-A as a standalone strategy in patients who fail to respond to CGRP-targeted therapies (i.e., less than 30% reduction in monthly headache days (MHDs) following treatment with CGRP-pathway-targeting mAbs) in economically restrictive scenarios or when pursuing monotherapy.

In the present real-world experience, we aim to investigate the effectiveness of BoNT-A in a cohort of individuals with resistant CM having previously failed at least three classes of oral repositioning drugs and at least one CGRP-mAb.

## 2. Results

The whole population consisted of 53 participants with CM (85% female, aged 48.6 ± 14.5 years, range 20–73). The average time since migraine onset was 21.52 ± 13.39 years, while the average age at CM development was 36.1 ± 15.7 years. Patients had previously failed a median of four [interquartile range (IQR) 1] treatments with repositioning drugs and at least one CGRP-mAb (20.7% erenumab, 47.1% fremanezumab, 41.5% galcanezumab and 1.8% eptinezumab). Six patients had failed two different CGRP-mAbs. The patient cohort showed at baseline a mean frequency of 21.64 (SD 5.65) headache days per month. Among the initial cohort of 53 patients, six were lost to follow-up: five at month 3 (three due to lack of efficacy and two because of intolerance to injection-related pain) and one at month 6 (due to lack of efficacy). Demographic and clinical features of disease severity of participants included in the study are reported in Table 1.

### 2.1. Primary Outcomes

**Figure 1:**
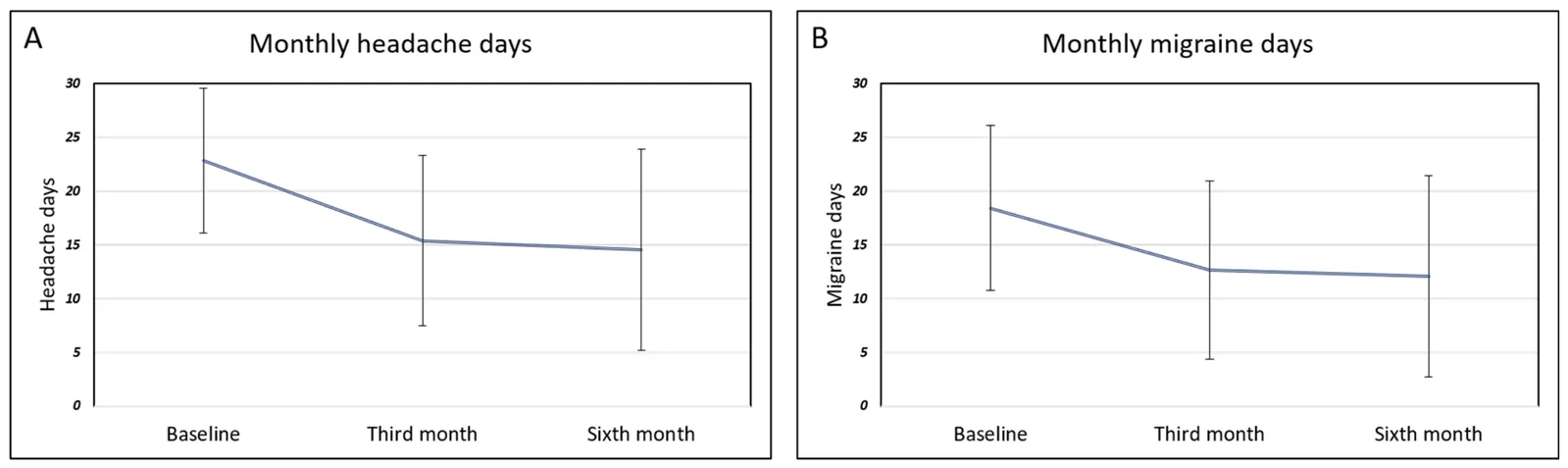
Mean monthly headache days (MHDs) (**A**) and monthly migraine days (MMDs) (**B**) at baseline, month 3, and month 6 following onabotulinumtoxinA treatment. Error bars represent standard deviations (SDs).

**Figure 2:**
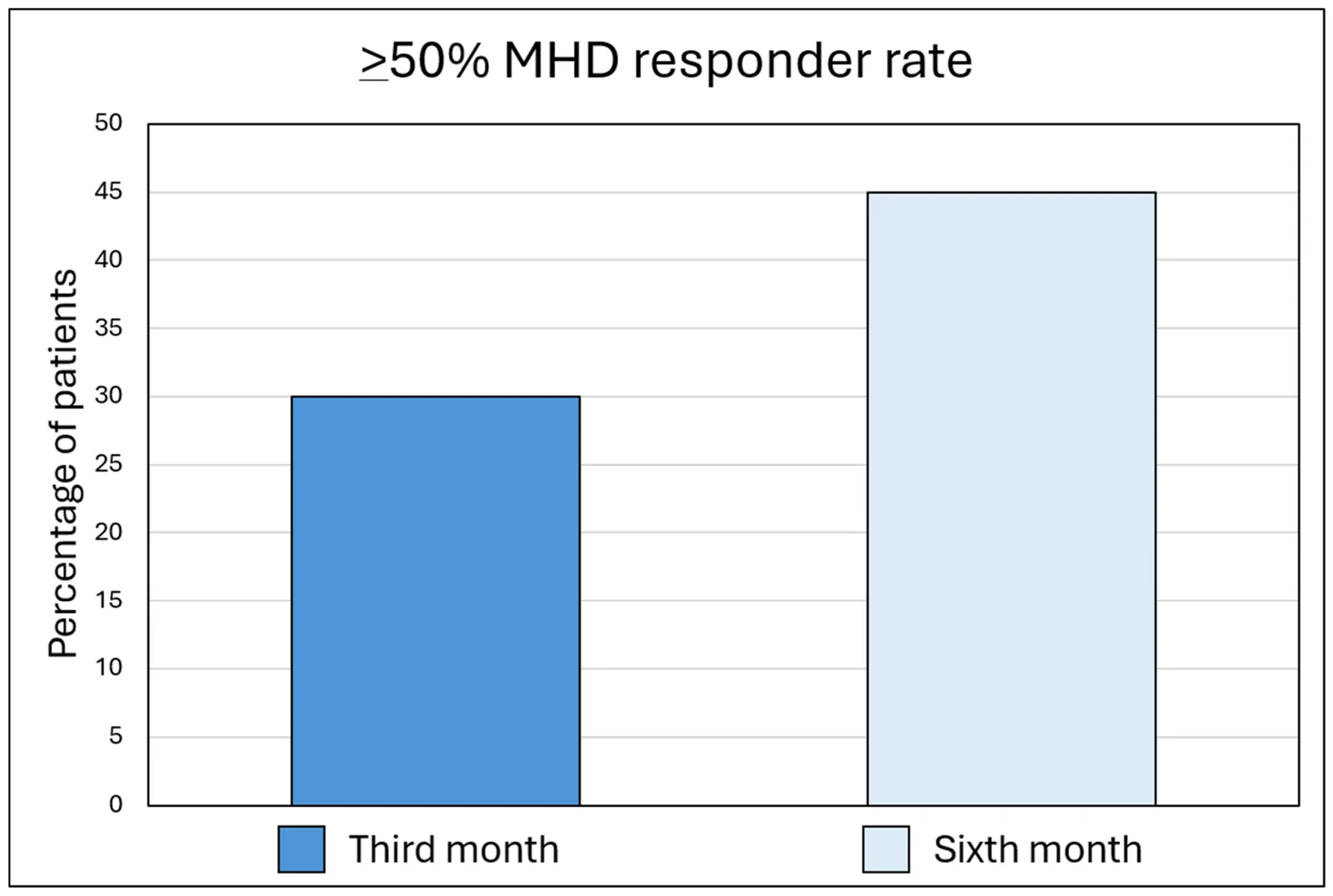
Proportion of patients achieving a ≥50% reduction in monthly headache days (MHD) at month 3 (T1) and month 6 (T2) following BoNT-A treatment initiation.

BoNT-A treatment was associated with a significant and progressive reduction in monthly headache days over the 6-month follow-up, with significant improvements already evident at month 3 and maintained at month 6 (Table 2; Figure 1). Consistently, the proportion of patients achieving a ≥50% reduction in monthly headache days increased over time (Figure 2).

### 2.2. Secondary Outcomes

**Figure 3:**
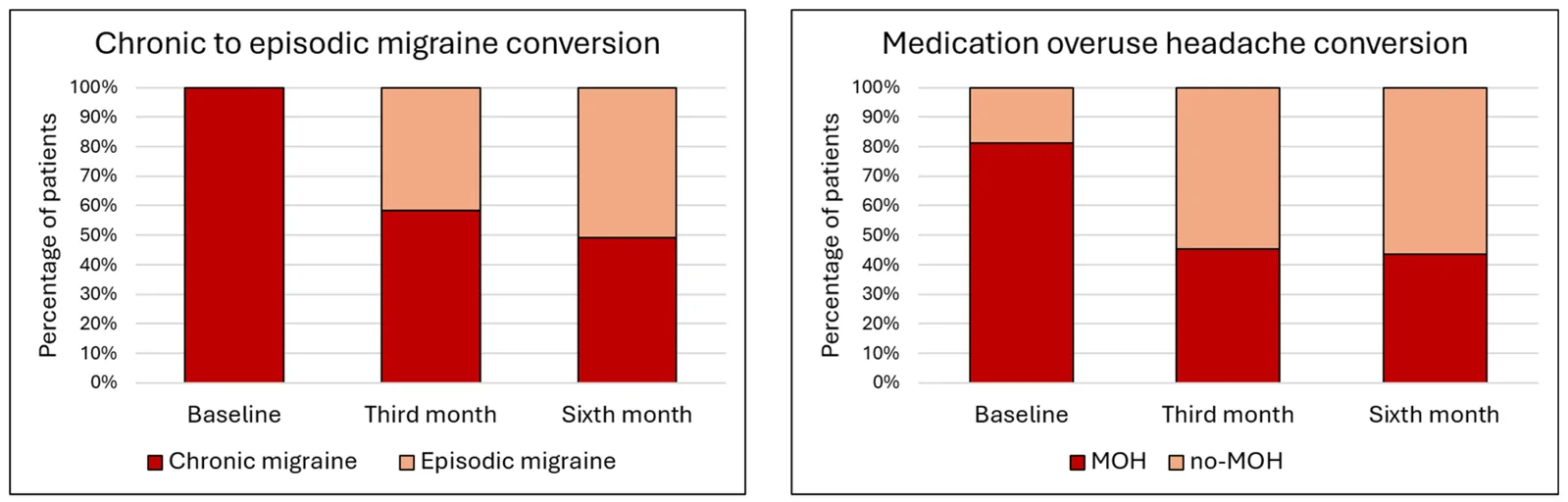
Percentage of patients with chronic migraine versus episodic migraine and with medication overuse headache (MOH) versus no-MOH at baseline, month 3, and month 6 following treatment initiation.

**Figure 4:**
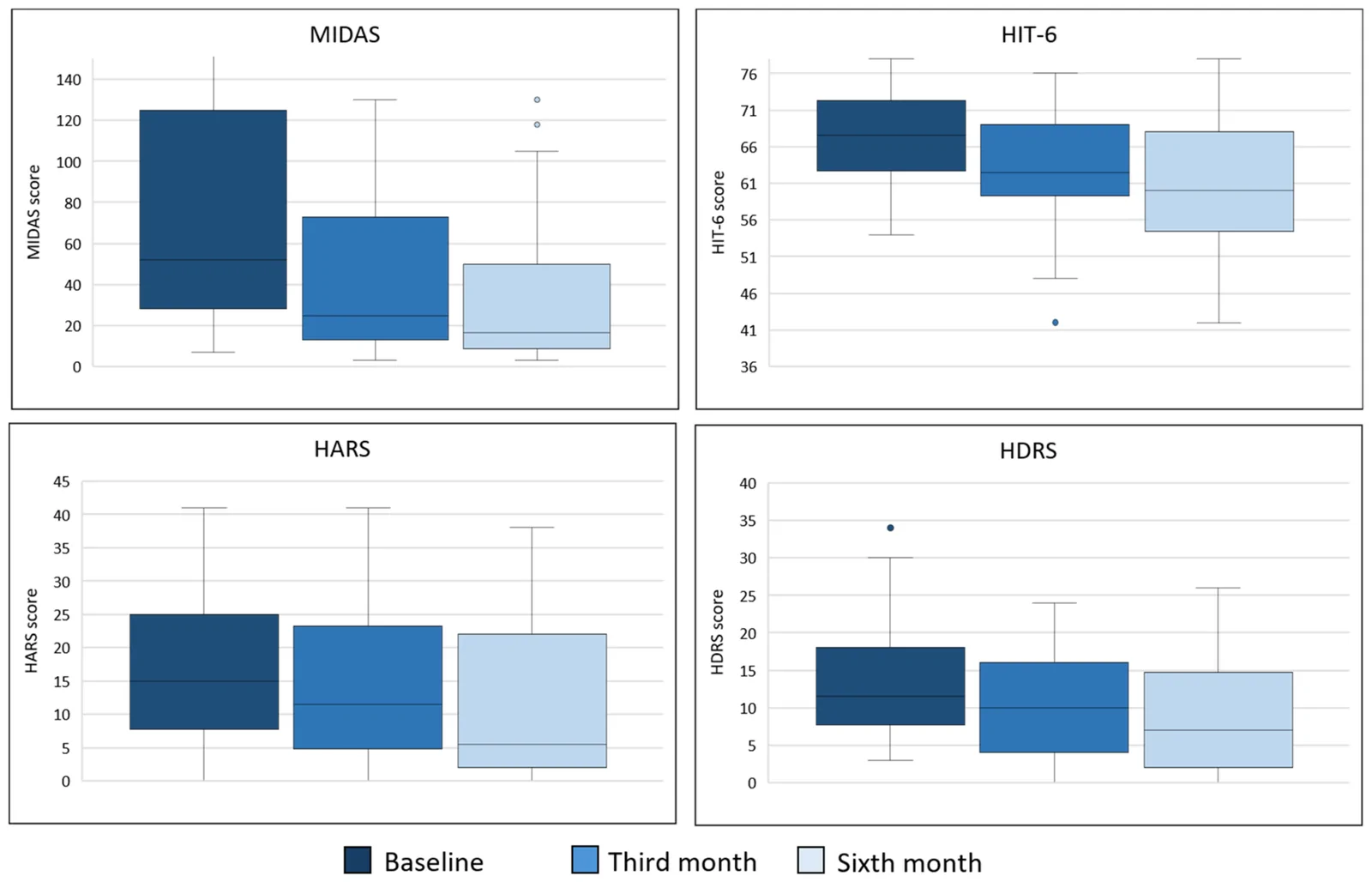
Changes in the evaluated patients’ reported and clinician-rated outcomes from baseline to month 3 and month 6 following treatment initiation. Data are presented as box-and-whisker plots showing the median, interquartile range (IQR), whiskers extending to 1.5 × IQR, and outliers.

**Figure 5:**
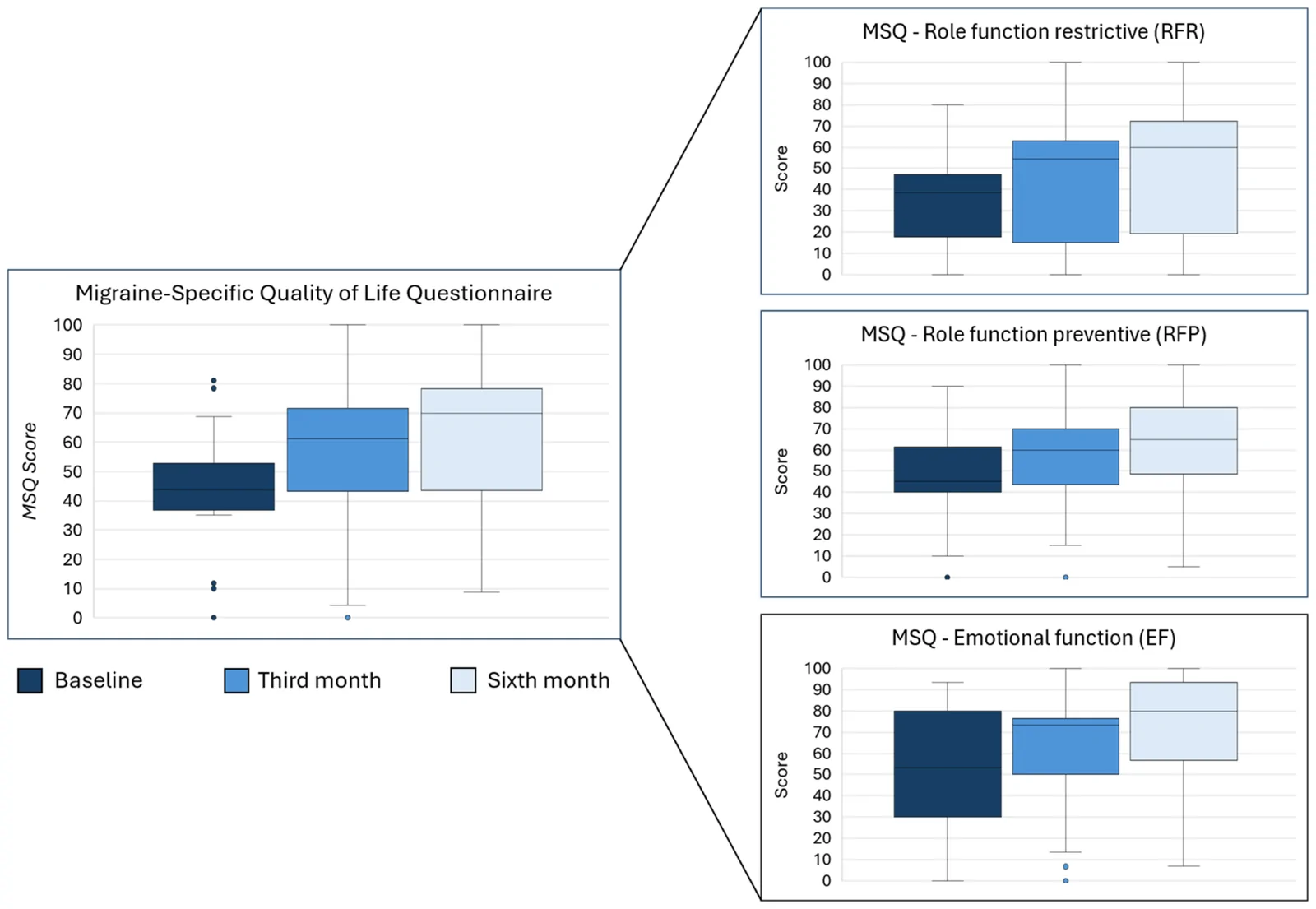
Longitudinal changes in Migraine-Specific Quality of Life Questionnaire version 2.1 (MSQ v2.1) total and subscale scores during the 6-month follow-up period. Data are presented as box-and-whisker plots showing the median, interquartile range (IQR), whiskers extending to 1.5 × IQR, and outliers.

Compared with baseline, patients showed significant improvements across all secondary clinical outcomes at both follow-up visits (Table 2). Monthly migraine days progressively decreased over the 6-month treatment period (Figure 1). In parallel, an increasing proportion of patients converted from chronic to episodic migraine, while the prevalence of medication-overuse headache was almost halved and remained consistently lower throughout follow-up (Figure 3). Migraine-related disability and headache impacts also improved significantly, as reflected by reductions in MIDAS and HIT-6 scores (Figure 4). Similarly, anxiety and depressive symptoms progressively decreased during treatment (Figure 4). Finally, migraine-specific quality of life significantly improved over time, with consistent benefits observed at both the third- and sixth-month assessments (Figure 5).

## 3. Discussion

The present real-world study suggests that BoNT-A represents an effective preventive option for patients with resistant CM who had previously failed at least three classes of oral preventive medications and one or more CGRP-mAbs. Notably, almost half of the cohort transitioned from chronic to episodic migraine during the 6-month follow-up, accompanied by a marked reduction in medication-overuse headache. In addition to reducing monthly headache frequency, treatment was associated with significant improvements in migraine-related disability and disease impact, as well as reductions in anxiety and depressive symptoms, ultimately translating to a better migraine-specific quality of life.

Nowadays, the therapeutic approach to patients with resistant CM remains particularly challenging, especially in those who have failed treatment with CGRP mAbs [20]. These individuals represent a highly difficult-to-treat subgroup, often characterized by a long disease duration, high attack frequency, significant disability, and multiple prior treatment failures [21]. In this context, management requires careful consideration of alternative strategies that extend beyond the selective blockade of the CGRP pathway [22]. Indeed, according to De Vries and colleagues, non-response may be related to insufficient blockade of the CGRP pathway: the CGRP ligand may bind and activate alternative receptors such as those for amylin or adrenomedullin; conversely, the CGRP receptor itself may be activated by different ligands such as amylin, depending on whether an anti-ligand or anti-receptor antibody is used [23]. In addition, alternative pathways, including PACAP and substance P, may represent predominant mechanisms driving migraine pathophysiology in certain patients. Finally, differences in drug delivery may also play a role, as BoNT-A is locally administered and taken up by trigeminal nerve terminals, whereas CGRP-mAbs are delivered systemically, potentially resulting in distinct pharmacokinetic and therapeutic effects.

In this scenario, our findings suggest that BoNT-A may be effective in achieving a substantial reduction in attack frequency, in terms of both MHD and MMD. Notably, a considerable proportion of patients converted from CM to episodic migraine during follow-up, with a parallel resolution of medication overuse observed in a high percentage of cases. These clinical improvements were associated with a reduction in migraine-related disability and overall disease burden. In particular, migraine impact, as assessed by the HIT-6, showed a median reduction of 6.5 points, exceeding the five-point threshold generally considered clinically meaningful [24]. Furthermore, treatment was associated with an improvement in quality of life, as measured by the Migraine-Specific Quality-of-Life Questionnaire version 2.1 (MSQ v2.1), with consistent benefits observed across all three domains, including restrictive role function, preventive role function, and emotional functioning. The proportion of patients achieving a ≥50% reduction in monthly headache days was consistent with previously published real-world evidence on onabotulinumtoxinA, including a meta-analysis reporting a pooled 50% responder rate of 46.6% at approximately 24 weeks [25].

The therapeutic effects of BoNT-A are believed to arise predominantly from modulation of peripheral trigeminovascular nociceptive signalling, reducing the activation of unmyelinated meningeal afferents and, consequently, diminishing the transmission of nociceptive inputs to central trigeminovascular neurons within the spinal trigeminal nucleus. More specifically, BoNT-A inhibits the exocytosis of vesicles containing not only CGRP but also other neuropeptides (including PACAP, VIP, glutamate and substance P, which are relevant in migraine pathophysiology), as witnessed by provocation studies demonstrating how the intravenous administration of these neuropeptides can trigger migraine-like attacks in susceptible patients [26,27,28,29,30,31,32,33]. Furthermore, BoNT-A has been shown to prevent the insertion of pain-sensitive ion channels (e.g., TRPV1, TRPA1) into the membranes of nociceptive neurons [34,35]. These pain-sensitive ion channels play a pivotal role in migraine pathophysiology by mediating nociceptive transmission and neurogenic inflammation within the trigeminovascular system. Moreover, their upregulation is involved in peripheral and even central sensitizations, which are well-known processes underlying the transition from episodic to chronic migraine [36]. Finally, BoNT-A preferentially prevents activation and sensitization of wide dynamic range (WDR) neurons acting on unmyelinated C-fibres, whereas CGRP mAbs predominantly modulate the activity of thinly myelinated Aδ-fibers [37,38]. Together, pre-clinical evidence further reinforces the notion that the two different therapeutic strategies may engage distinct neuronal substrates, corroborating, on the other hand, the putatively complementary mechanisms of action of both BoNT-A and CGRP-signalling neutralizing drugs [37,38,39,40,41,42]. Although the concomitant use of BoNT-A and CGRP-mAbs has been associated with additive, and in some cases potentially synergistic, clinical benefits, the present study was not designed to compare combination therapy with BoNT-A monotherapy. Therefore, no conclusions can be drawn regarding the superiority of one strategy over the other. Nevertheless, our findings suggest that BoNT-A should also be considered as a standalone option in patients who show an inadequate response to CGRP-mAbs [39,40,41,42]. These findings could be of particular interest in economically restrictive scenarios, when pursuing monotherapy or when the reduction in MMD remains below a clinically meaningful threshold (e.g., <30% reduction in MMD). In this case, data on the likelihood that combination therapy will yield a substantial incremental benefit compared to single treatments alone is still limited. In such cases, switching strategy rather than layering treatments may be considered. Conversely, in patients who achieve a partial but clinically relevant response to CGRP mAbs (e.g., ≥30% reduction in MMD), the addition of BoNT-A may be more justified, as it can capitalize on complementary mechanisms of action. Therefore, a combined approach could be more coherent when there is an existing, albeit minimal, therapeutic signal, whereas it is less compelling in the context of an absent response to CGRP-mAbs therapy. Furthermore, as CGRP-mAbs are increasingly introduced after failure of oral preventive therapies, it can be argued that BoNT-A should be reserved for patients who do not respond to CGRP-mAbs, given the longer time often required to adequately assess its therapeutic benefit.

A major strength of this study lies in the relatively homogeneous study population, which was composed exclusively of patients with chronic migraine. This reduced clinical heterogeneity and allowed a more reliable assessment of the effectiveness of BoNT-A within a well-defined and clinically relevant population, supporting the applicability of the findings to patients with similar disease characteristics. Moreover, by specifically enrolling individuals with multiple previous preventive treatment failures, this study provides clinically relevant evidence in a particularly difficult-to-treat subgroup for whom therapeutic options remain limited. Nevertheless, several limitations should be acknowledged. First, the observational, non-randomized, open-label design did not include either a placebo or an active comparator group. Consequently, the findings should be interpreted with appropriate caution, as the influence of residual confounding and other uncontrolled factors cannot be completely excluded. Secondly, in the absence of a washout period between the end of CGRP-mAbs treatment and the initiation of BoNT-A administrations, it cannot be entirely ruled out that the observed effectiveness may reflect a combined effect of residual antibody activity (i.e., wind-up phenomenon) together with BoNT-A, rather than BoNT-A alone. However, the sustained clinical benefit observed after six months of continuous BoNT-A treatment, well beyond the expected duration of action of CGRP-mAbs, strongly supports a direct therapeutic effect attributable to BoNT-A. Although categorical efficacy outcomes were analysed using a conservative non-responder imputation approach, continuous outcomes were evaluated using complete case analyses. Therefore, a modest overestimation of treatment effectiveness for these continuous measures cannot be excluded, particularly in patients who discontinued follow-up because of a lack of efficacy. Finally, since only six patients had previously failed more than one CGRP monoclonal antibody, it cannot be excluded that some patients might have responded to an alternative CGRP-targeted therapy, limiting mechanistic inferences regarding the effectiveness of BoNT-A after CGRP-mAb failure.

## 4. Conclusions

In conclusion, our findings support BoNT-A as a viable option after CGRP-mAb failure in patients with resistant chronic migraine, likely due to its broader and mechanistically distinct action on peripheral nociceptive pathways.

## 5. Materials and Methods

### 5.1. Study Design and Participants

This prospective, observational, non-randomized multicentre study was conducted to evaluate the effectiveness and tolerability of quarterly BoNT-A as a preventive treatment in patients with resistant CM [43,44]. Eligible participants had previously failed at least three classes of oral preventive medications, as required by regulatory and reimbursement criteria for access to CGRP-mAbs and at least one CGRP-mAb. Adults (≥18 years) fulfilling the ICHD-3 diagnostic criteria for CM [1.3 ICHD-3] were consecutively enrolled. CM was defined as headache occurring on ≥15 days per month for more than 3 months, with migraine features present on at least 8 days per month [45]. The diagnosis was established by a neurologist with expertise in headache disorders using a semi-structured clinical interview. Patients were eligible if they had discontinued at least three oral preventive treatments and one CGRP-mAb because of either insufficient efficacy or intolerable adverse events, with no restriction on the maximum number of previous preventive failures. Lack of efficacy was defined as a <30% reduction in MHD following treatment with standard preventive medications (repositioning drugs) administered at an adequate dose for at least 6 weeks and/or after at least 3 months of treatment with a subcutaneous CGRP-mAbs, according to the European Headache Federation (EHF) criteria [37]. Tolerability failure was defined as permanent treatment discontinuation due to adverse events at any time during therapy. All participants were followed for 6 months. They initially received the standard PREEMPT fixed-site/fixed-dose protocol (155 U) [20,46]. An additional 40 U were subsequently administered according to the PREEMPT “follow-the-pain” paradigm, resulting in a total dose of 195 U. The additional injections were distributed within the temporalis (5 U per side), occipitalis (5 U per side), and upper trapezius muscles (10 U per side), in accordance with the original PREEMPT protocol. Other oral migraine-preventive medications (alone or in combination) at a stable dose were allowed concomitantly with BoNT-A injections; however, gepants were not permitted during the study.

### 5.2. Collected Variables

At baseline (T0), demographic and clinical data were collected, including sex, age, body mass index (BMI), migraine history (age at migraine onset, age at migraine chronification, and comorbidities), and previous preventive treatment failures involving topiramate, amitriptyline, venlafaxine, candesartan or lisinopril, beta-blockers, flunarizine, and CGRP-mAbs.

Clinical assessments were performed at baseline (T0), month 3 (T1), and month 6 (T2) following BoNT-A treatment. The collected outcome measures included (i) headache frequency, expressed as MHD and MMD; (ii) the presence of medication-overuse headache (MOH); (iii) migraine-related disability, assessed using the Migraine Disability Assessment (MIDAS) questionnaire [47,48]; (iv) migraine-related impact, assessed with the Headache Impact Test (HIT-6) [49,50]; (v) anxiety and depressive symptoms, evaluated using the Hamilton Anxiety Rating Scale (HARS) and Hamilton Depression Rating Scale (HDRS) [51,52]; and (vi) migraine-specific quality of life, assessed with the MSQ v2.1 [53].

Participants were instructed to prospectively complete a paper headache diary throughout the study. A migraine day was defined as any day on which a headache fulfilled migraine characteristics or responded to triptan treatment, whereas a headache day was defined as any day with headache, irrespective of whether the clinical features were consistent with migraine or tension-type headache. Each patient gave free, informed consent for participation in the study and the analysis and publication of the protocol data.

The study was approved as part of the Registro Italiano Cefalee (RICe) study by the local ethics committee (Studio RICe, 14591_oss CEAVC Studio RICe, 14591_oss and subsequent amendments). The online open database Research Electronic Data Capture (REDCap) and the Empedocle electronic platform (developed for the RICe study) were used for data collection.

### 5.3. Primary Outcomes

The co-primary effectiveness outcomes of the present real-world observation were

(i)  Change in MHD from baseline to the third and sixth months;
(ii) Percentage of responders (participants who achieved at least 50% reduction from their individual baseline in MHD) after the third and sixth months of preventive treatment with BoNT-A.

### 5.4. Secondary Outcomes

Secondary outcomes included the evaluation from baseline to the third and sixth months of

(i)     Change in MMD;
(ii)    Percentage of patients converting from chronic to episodic migraine;
(iii)   Percentage of patients reverting from MOH;
(iv)   Changes in migraine-related disability (evaluated by MIDAS);
(v)    Changes in migraine-related impact (evaluated by HIT-6);
(vi)   Changes in anxious symptoms (evaluated by HARS);
(vii)  Changes in depressive symptoms (evaluated by HDRS);
(viii) Changes in migraine-related quality of life and functioning (evaluated by MSQ 2.1).

### 5.5. Statistical Analysis

The distribution of continuous variables was assessed for normality using the Shapiro–Wilk test before selecting the appropriate statistical approach. Normally distributed variables are presented as mean ± standard deviation (SD), whereas non-normally distributed variables are summarized as median and interquartile range (IQR). Categorical variables are reported as counts and percentages. Comparisons of normally distributed continuous variables were performed using the paired Student’s *t*-test, whereas the Wilcoxon signed-rank test was used for variables not meeting the assumption of normality. Changes in paired categorical variables across follow-up were assessed using the McNemar test. All statistical tests were two-sided, and statistical significance was defined as a *p*-value < 0.05. Bonferroni correction was applied to account for multiple comparisons. Analyses were primarily performed on available cases. Patients lost to follow-up were reported with reasons for discontinuation. To minimize the risk of overestimating treatment effectiveness, the proportions of patients achieving a ≥50% reduction in MHD, converting from chronic to episodic migraine, and resolving medication-overuse headache were calculated on the entire baseline cohort of 53 patients. Participants lost to follow-up were conservatively considered non-responders and classified as remaining with chronic migraine and medication-overuse headache (non-responder imputation). Continuous efficacy outcomes were analysed using available follow-up data (complete-case analysis). Missing data were limited to some patient-reported outcome measures, for which analyses were performed on available cases without data imputation. All statistics were performed using STATA version 16 and Statistical Package for Social Science version 20 (SPSS, Chicago, IL, USA).

## Figures and Tables

**Table 1:** Demographic and clinical features of patients.

Number of participants	53
Female (n)	46
Age (mean and SD)	48.6 ± 14.5
BMI (mean and SD)	24.12 ± 3.50
Age at migraine onset (mean and SD)	21.52 ± 13.39
Age at migraine chronification (mean and SD)	36.14 ± 15.78
Prior treatment failures (median and IQR)	4 (1)
Previous SoC failures	
Tricyclic antidepressants	46 (86.8)
SSRI/SNRI	15 (34.9)
Beta-blockers	36 (67.9)
Antiepileptic drugs	38 (71.7)
Calcium channel blockers	27 (50.9)
Prior failure with CGRP-mAbs n (%)	
Eptinezumab n (%)	1 (1.9)
Erenumab n (%)	11 (20.8)
Fremanezumab n (%)	25 (47.2)
Galcanezumab n (%)	22 (41.5)

BMI: body mass index; SSRI: Selective Serotonin Reuptake Inhibitor; SNRI: Serotonin–Norepinephrine Reuptake Inhibitor. n: number; IQR: Interquartile Range. SD: standard deviation.

**Table 2:** Change in clinical outcomes from baseline to third month and sixth month in the 53 participants who completed the 6-month follow-up.

Outcomes	Baseline	Timeline	
		Third Month	Sixth Month
MHDs (mean ± SD) ^†^	22.83 ± 6.74	15.38 ± 7.91 ***	14.55 ± 9.35 ***
≥50% Reduction in MHD n (%)		16 (30)	24 (45)
MMDs (mean ± SD) ^†^	18.42 ± 7.66	12.65 ± 8.25 ***	12.06 ± 9.34 ***
MIDAS (median ± IQR) δ	52 (77)	24.5 (55.25) ***	16.5 (40.75) **
HIT-6 (median ± IQR) δ	67.5 (9)	62.5 (8.75) ***	60 (12.5) ***
HARS (median ± IQR) δ	15 (16.75)	11.5 (17.75) **	5.5 (19.25) ***
HDRS (median ± IQR) δ	11.5 (10)	10 (11.5) ***	7 (12.5) ***
MSQ v2.1 (median ± IQR) δ	42.94 ± 22.18	57.10 ± 32.94 ***	62.38 ± 32.66 ***
Role function restrictive δ	37.14 ± 31.43	54.29 ± 40.71 **	60 ± 42.14 **
Role function preventive δ	45 ± 25	60 ± 23.75 **	65 ± 23.75 **
Emotional function δ	53.33 ± 56.67	66.67 ± 38.33 *	73.33 ± 38.33 **
Conversion from CM to EM n (%)		22 (41.5)	27 (50.9)
MOH n (%)	43 (81.1)	24 (45.3)	23 (43.4)

CM: chronic migraine; EM: episodic migraine; MMDs: monthly migraine days; MHDs: monthly headache days; MOH: medication overuse headache; MIDAS: Migraine Disability Assessment; HIT-6: Headache Impact Test-6; HARS: Hamilton Anxiety Rating Scale; HDRS: Hamilton Depression Rating Scale; MSQ: Migraine-Specific Quality-of-Life Questionnaire; n: number; IQR: Interquartile Range. SD: standard deviation; * *p* < 0.05, ** *p* < 0.01, *** *p* < 0.001 statistically significant (in the comparison with baseline). ^†^ Paired *t*-Test, δ Wilcoxon signed-rank test.

## Data Availability

The data presented in this study are available on request from the corresponding author due to privacy and ethical restrictions, as they consist of anonymized patient-level clinical data collected during routine clinical practice.
